# Molecular analysis of two local falciparum malaria outbreaks on the French Guiana coast confirms the *msp1 *B-K1/*varD *genotype association with severe malaria

**DOI:** 10.1186/1475-2875-4-26

**Published:** 2005-06-21

**Authors:** Eric Legrand, Beatrice Volney, Anne Lavergne, Caroline Tournegros, Loïc Florent, Doris Accrombessi, Micheline Guillotte, Odile Mercereau-Puijalon, Philippe Esterre

**Affiliations:** 1 Centre National de Référence sur la Chimiorésistance du Paludisme dans la région Antilles – Guyane, Institut Pasteur de la Guyane, BP 6010, F-97306 Cayenne-Cedex, France; 2 Unité d'Immunologie Moléculaire des Parasites, Unité de Recherche Associée 2581 du Centre National de la Recherche Scientifique, Institut Pasteur, Paris, France

## Abstract

**Background:**

*Plasmodium falciparum *outbreaks can occur in the coastal area of French Guiana, where the population is essentially non-immune. Two sporadic outbreaks were observed, including one with severe malaria cases. To characterize these outbreaks and verify previous observations of specific genotype characteristics in severe malaria in this area, all cases from each outbreak were studied.

**Methods:**

*P. falciparum *genotypes for six genetic loci were determined by PCR amplification from peripheral blood parasites. The *msp1/*block2 and *msp2 *genotypes were determined by DNA sequencing. Microsatellite and *varD *genotyping was based on size polymorphism and locus-specific amplification.

**Results:**

The outbreak including severe malaria cases was associated with a single genotype. The other mild malaria outbreak was due to at least five distinct genotypes.

**Conclusion:**

Two distinct types of outbreak occured despite systematic and sustained deployement of malaria control measures, indicating a need for reinforced vigilance. The *varD*/B-K1 *msp1 *linkage and its association with severe malaria in this area was confirmed.

## Background

The annual malaria incidence in French Guiana has increased ten-fold during the last 30 years, reaching nowadays approximately 3%, with 60% due to *Plasmodium falciparum *and 40 % due to *Plasmodium vivax*. French Guiana is an area with multidrug resistant *P. falciparum *malaria. Transmission occurs in isolated foci located in settlement pockets within the Amazonian forest and along the rivers. The main malaria-endemic areas are located along the Maroni and Oyapock rivers, which serve as natural frontiers with Surinam and Brazil, respectively [1]. As a result of three-monthly insecticide spraying campaigns, there are virtually no malaria cases along the coast, where 80% of the population resides. However, outbreaks, which usually are of short duration and affect a small number of patients, are occasionally reported in the coastal area.

The *P. falciparum *parasite population of French Guiana presents a remarkably low degree of polymorphism, with a clonal type structure [2]. The parasite diversity is so restricted that the circulation of specific genotypes could be followed within the area, making it possible to investigate the clinical impact of specific parasite genotypes. A comparative analysis of parasite genetic characteristics in isolates collected from mild and severe malaria patients has highlighted a significant linkage disequilibrium between a particular *msp1*/block2 allele that was called B-K1 and a particular *var *gene called *varD *in isolates from patients with severe *P. falciparum *malaria [3].

To verify this association and better understand the possible cause of local outbreaks in the coastal area located away from the main endemic sites, two *P. falciparum *outbreaks were studied, one with five cases, including two cases of severe malaria and one death, the other with nine mild malaria cases. The isolates from each case were characterized for both outbreaks using a six-locus genotyping approach, including *varD, msp1*/block2, *msp2 *[2-7], as well as three microsatellite loci [8].

## Methods

### Parasite isolates and patient information

**Figure 1:**
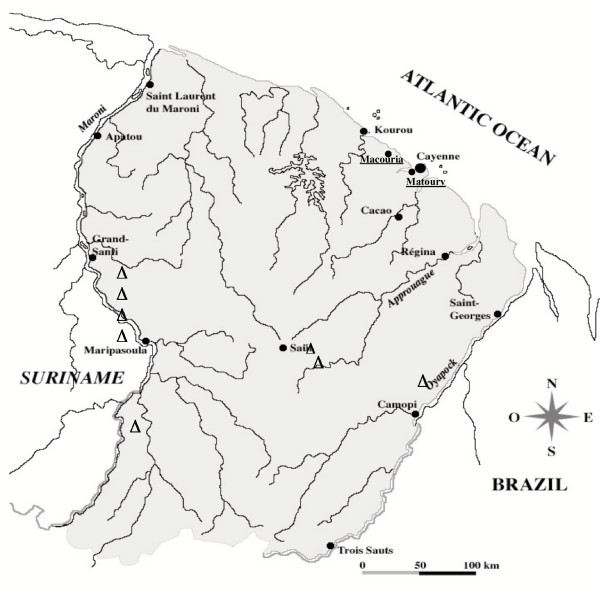
Map of French Guiana. Triangles indicate the localization of the severe malaria cases with K1-B/*var D *described by Ariey et al. [3]. The towns of Macouria and Matoury are indicated.

The outbreaks occurred in Macouria and Matoury, both located on the French Guiana coast, an area with unstable, low transmission, away from the main endemic areas along the Maroni and the Oyapock (Fig. 1). The international airport close to Cayenne, the main city, is in Matoury. The isolates were collected from patients with *P. falciparum *malaria in health centres and sent to the laboratory for routine drug susceptibility phenotyping and genotyping analysis. The policy of the malaria control services is to get an *in vitro *susceptibility profile for each diagnosed *P. falciparum *malaria case. To this end, a 5 mL blood sample was systematically collected by venepuncture in EDTA tubes and maintained at 4°C during shipment to the laboratory. The samples were immediately processed for *in vitro *susceptibility assays and a 0.5 mL aliquot was frozen at -20°C for subsequent DNA preparation. The five Macouria isolates were collected in June and July 2001 from three mild (E57, E64 and E72) and two severe cases (E61, a fatal case, and E62). Patient E57 had visited the endemic Macapa region in Brazil one month before the malaria attack. The other four Macouria patients did not report having left the area. The nine Matoury isolates, collected from February to June 2002 originated from mild malaria cases, none of whom reported a recent visit to an endemic area.

### DNA extraction and genotyping

**Figure 2:**
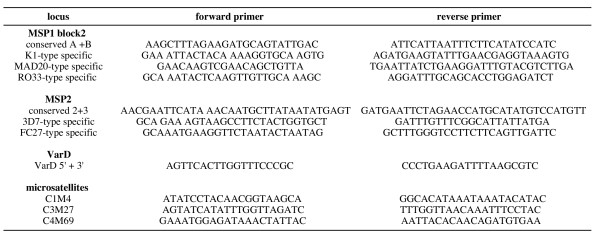
Sequence of primers.

DNA was extracted as described [4]. Genotyping was performed by PCR using the primers listed in Figure 2. For *msp1 *block2, *msp2 *and *varD*, PCR was performed in a 50 μL final volume containing the DNA template, 1.5 mM MgCl_2_, 2 μM each primer, 200 μM dNTP, and 1.5 units of AmpliTaq Gold polymerase (Applied Biosystems, Foster City, CA, USA). The PCR conditions for *msp1 *block2 and *msp2 *were : one cycle at 95°C for 5 min, followed by 40 cycles at 94°C for 1 min, 56°C for 2 min, 72°C for 2 min, and final extension at 72°C for 10 min. The *varD *PCR conditions were: one cycle at 94°C for 5 min, followed by 35 cycles at 94°C for 10 sec, 61°C for 1 min 30 sec, 72°C for 2 min, and one cycle at 72°C for 10 min. The PCR products were analysed by agarose gel electrophoresis and directly sequenced (Qbiogen, Every, France). Sequences were aligned using the Clustal W program, followed by manual analysis using the ED editor of the MUST package [9].

Microsatellite typing was done using the C1M4, C3M27 and C4M69 loci, located on chromosomes 1, 3 and 4, respectively. The locus-specific primers described by Su *et al*. [8] were used (see Figure 2). Amplification was done in 15 μL reaction volume containing the DNA template, 1.5 mM MgCl_2_, 2 μM each primer, 200 μM each dNTP, and 0.5 Units *Taq *polymerase (Promega, Madison, WI, USA). The samples were subjected to 1 cycle at 94°C for 7 min, followed by 35 cycles at 94°C for 30 sec, 52°C for 30 sec, 47°C for 30 sec, 72°C for 30 sec, and a final extension step at 72°C for 15 min. The PCR products were analysed by 3% Metaphor agarose gel (FMC Bioproduct, Rockland, Maine, USA) electrophoresis and stained with ethidium bromide. Fragment size was calculated using the Taxotron software (P.A.D. Grimont, Institut Pasteur, Paris).

## Results and discussion

**Figure 3:**
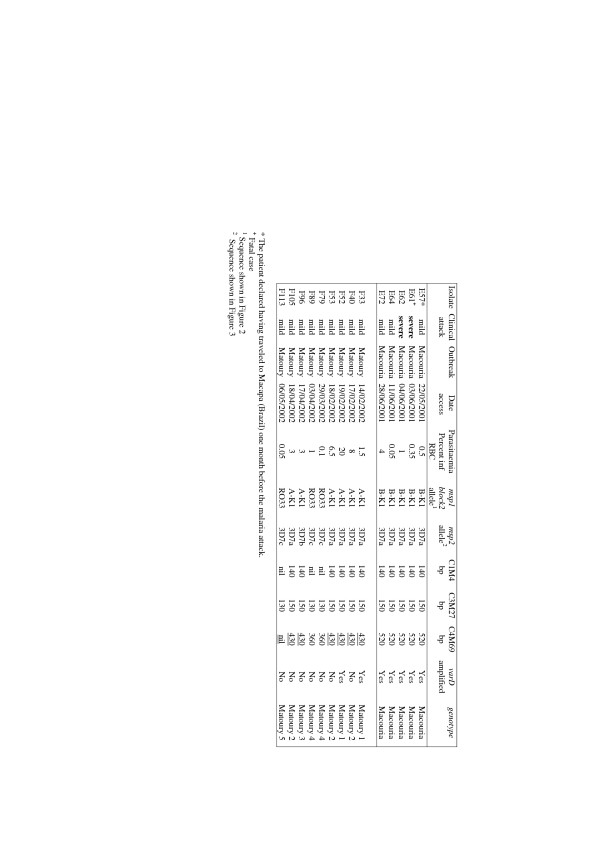
*msp1*/block2, *msp2*, microsatellite and *varD P. falciparum *genotypes observed in the Macouria and Matoury outbreaks.

**Figure 4:**
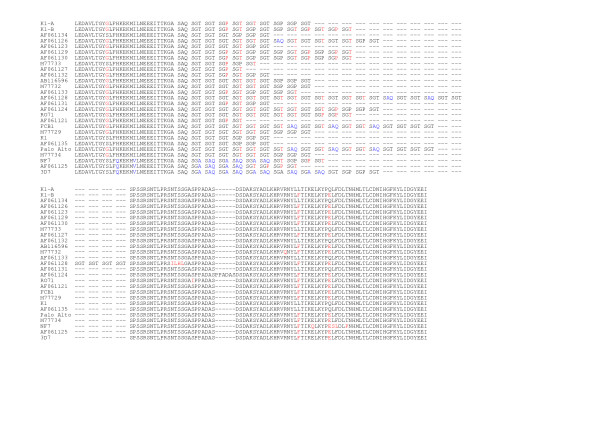
Deduced amino acid sequence of the French Guiana A-K1 and B-K1 *msp1/*block2 alleles and alignment with alleles described in other areas. Single letter codes are used for amino acids. The name of the reference laboratory strains are indicated. The accession number for K1, FCB1, Palo Alto, NF7 and RO71 were X03371, AF286876, M37213, M19144, X61930. For 3D7, the sequence of the genome sequence (locus PFI1475w) is shown. For field isolates the Genbank accession number was used. The reference sequence used was K1. Deletions are indicated by a dash and mutant residues are in red, except for the 3D7-types which are in blue.

PCR amplification of the *msp1/block2 *and *msp2 P. falciparum *loci generated a single band for all isolates (Fig. 3). Each PCR band was sequenced. This showed three distinct alleles for each locus. All Macouria samples carried the same 464-bp *msp1 *block2 allele, which belonged to the K1-type allelic family, corresponding to the allele called B-K1 in a previous study [3]. A smaller (431 bp) K1-type allele, previously called A-K1 [3], and a 398 pb RO33-type allele were observed in six and three Matoury isolates, respectively. All three RO33-type sequences were identical to the RO33 allele (accession number Y00087). Alignment of the B-K1 and A-K1 deduced protein sequence with sequences available in the databases identified several alleles observed in other regions of the world showing up to 99% and 97% identity, respectively. However, no isolate in the database had precisely similar tri-peptide repeats as B-K1 or A-K1. This is in line with recent evidence indicating that *msp1/*block 2 (and *msp2) *repeats evolve at a faster rate than SNPs in the non-repeated domains [10]. Numerous isolates showed in addition single nucleotide polymorphisms in the non-repeated sequences (Fig. 4).

**Figure 5:**
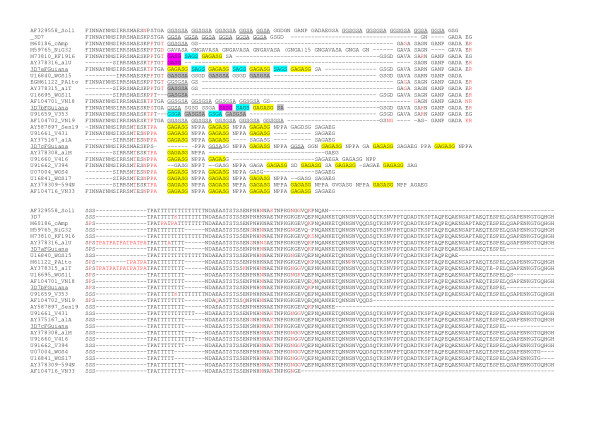
Deduced amino acid sequence of 3D7-a,-b and -c *msp*2 alleles and alignment with alleles described in other areas. Single letter codes are used for amino acids. The name of the reference laboratory strains are indicated. The 3D7 sequence (locus PFB0300c) was used as reference sequence. For field isolates the Genbank accession number was used. The Genbank accession number for Palo Alto was M61122. Deletions are indicated by a dash and mutant residues as compared to 3D7 are in red. The various repeat sequence types are underline or color-coded.

All three *msp2 *alleles belonged to the 3D7-type family and were arbitrarily called 3D7a (591 bp), b (561 bp) and c (603 bp). The 3D7a allele was observed in all five Macouria isolates and in five of nine Matoury isolates. The 3D7b and 3D7c allele were observed in one and three Matoury isolates, respectively (Fig. 3). Alignment of the deduced protein sequence showed that allele 3D7c clustered with a specific group of isolates showing typical particularities in the repeat region in particular the NPPA tetrapeptide interspersed within G-, A-, S- rich repeats, mainly GAGASG (Fig. 5). However, the 3D7c allele was peculiar within this group in having the largest number of both types of repeats NNPA and presenting GGSA repeats as well. The 3D7a and 3D7b also presented each a specific arrangement of repeats as well as SNPs in the non repeated regions.

To further explore the genetic make-up of the *P. falciparum *isolates, three microsatellite loci were studied. A single 140-bp C1M4 product was observed for all isolates, except F79, F89 and F113, where no band was visualized (Fig. 3). A 150-bp C3M27 product was amplified from all Macouria isolates and from the F33, F40, F52, F53, F96 and F105 Matoury isolates. A distinct 130-bp C3M27 allele was detected in isolates F79, F89 and F113. For the C4M69 locus, a single 520-bp band was obtained for all Macouria isolates, a 430-bp band for Matoury isolates F33, F40, F52, F53, F96 and F105 and a 360-bp fragment for the Matoury isolates F79 and F89. No product was obtained for this locus from isolate F113 (Fig. 3). This data indicated that for all five single copy loci investigated, all isolates generated a single, monomorphic PCR fragment. We can thus reasonably consider them as being monoclonal infections. This confirms previous findings in this area, where a very high percentage of single infections was observed [2,3], and is in line with reports from other areas of South America [5,6].

French Guiana is a hypoendemic area, where the population has limited, if any, immunity. There are between 6,000–7,000 mild malaria cases and only between four and 20 severe malaria cases each year [1,3]. Previous studies have highlighted specific genotype characteristics in *P. falciparum *isolates from severe cases in this setting, with the B-K1 *msp1 *allele in strong linkage disequilibrium with a particular *var *gene, called *varD *[3]. The *var *genes code for the variant adhesins that mediate the cytoadhesion of parasitized erythrocytes to specific host receptors that undoubtedly contribute to severe malaria. As highlighted in Figure 3, all five Macouria isolates, including the two severe cases, had the same 5-loci genotype, suggesting that a single virulent isolate was present. These isolates were also tested fo the presence of the *varD *gene. A typical *varD *product was amplified from all five Macouria isolates. Only two Matoury isolates (isolates F33 and F52) carried the *varD *locus, none of which harboured the B-K1 *msp1 *allele (Fig. 3). Overall, the same genotype was observed in all Macouria isolates while there were at least five distinct genotypes in Matoury. The Matoury 1 and 2 genotypes differed at the *varD *locus. The Matoury 2 and Matoury 3 genotypes had a different *msp2 *allele. Furthermore, isolates F40 and F96 had a different drug susceptibility profile (data not shown). The Matoury 4 and Matoury 5 genotypes differed by the C4M69 microsatellite locus.

These data confirm the *varD*/B-K1 *msp1 *linkage and its association with severe malaria in this area. It is important to note that this association was previously detected in isolates collected in 1994-6, more than six years before the Macouria epidemic. Such a stability over time in a species with a high recombination rate [8] is consistent with previous data pointing to a high selfing rate in this area [3]. The severe malaria cases studied here originated from a geographic area quite distinct from the previous cases reported where the *varD*/B-K1 association was observed, as illustrated in Figure 1. As observed previously [3], the *varD*/B-K1 *msp1 *association was not strictly specific for severe malaria, as the same genotype was also observed in the non-severe Macouria cases. Whether the patients with mild malaria received an earlier treatment compared to those with severe malaria or were less susceptible due to their genetic or immune make up is unclear. The occurrence of two severe cases at one day interval in patients infected with this strain suggests a particular inherent virulence. Whether this is due to the particular *msp1 *and/or *varD *allele present is unknown. It may reflect physical association with another locus implicated in virulence. It is worth noting that isolate F52, which harboured *varD *together with the A-K1 *msp1 *allele, was collected from a patient with very high peripheral parasitaemia (20%), also considered a sign of severity [11]. *VarD *is one of approximately 60 members of the *P. falciparum var *genomic repertoire [12]. The presence of a particular *var *gene is not synonymous with its expression. Expression of *varD *was demonstrated in a patient with fatal *P. falciparum *malaria in a previous study [3], but could not be studied here. The data indicate that future investigations on *varD *expression in severe and non-severe malaria are warranted. Work is in progress to characterize the full *varD *gene sequence.

The different genetic profiles of the isolates involved in the two outbreaks reveal distinct onset and dynamics scenarios. Genotyping strongly suggests that the Macouria outbreak was due to one single parasite strain, but the origin of this strain is uncertain. Transmission from E57, the first registered case, to the other cases is unlikely, due to the short delay between the clinical attack and the attack experienced by E61, E62 and E64 (12, 13 and 20 days later, respectively). E67 may possibly have subsequently transmitted the strain to E72, who experienced a malaria attack 37 days later. The diversity of the Matoury isolates indicates that the outbreak was certainly caused by several distinct strains. Matoury accommodates the largest airport in the country and consequently may serve as an occasional transmission focus for parasites originating in neighbouring endemic malaria areas. The possible cause and mode of transmission in this city at that time have not been identified.

## Conclusion

The results point to two distinct types of outbreak in a region where malaria control measures are systematically deployed and sustained. Reinforced vigilance and rapid case notification are needed to ensure rapid deployment of vector control and personal protection measures to prevent such sporadic epidemics. The Macouria outbreak provoked two severe cases, including one death, a rare event in this area, where health facilities are well-equipped and treatment policy is regularly updated. Parasite genotyping confirmed the association of the B-K1 *msp1/varD *genotype with severe malaria, reinforcing the notion that some *P. falciparum *strains might cause more severe infections than others.

## Authors' contributions

A. Lavergne and E. Legrand did the sequences analysis. B. Volney, C. Tournegros, D. Accombressi and L. Florent did the laboratory experiments. M. Guillotte and O. Mercereau-Puijalon adapted the microsatellite typing to field isolates. O. Mercereau-Puijalon did the database search. All authors participated in the analysis and interpretation of data. E. Legrand and O. Mercereau-Puijalon wrote the manuscript.
